# Cutaneous Involvement of Extranodal NK/T Cell Lymphoma, Nasal Type, a Clinical and Histopathological Mimicker of Various Skin Diseases

**DOI:** 10.3390/dermatopathology9030037

**Published:** 2022-09-09

**Authors:** Preeyawat Ngamdamrongkiat, Sanya Sukpanichnant, Manasmon Chairatchaneeboon, Archrob Khuhapinant, Panitta Sitthinamsuwan

**Affiliations:** 1 Department of Pathology, Faculty of Medicine Siriraj Hospital, Mahidol University, Bangkok 10700, Thailand; 2 Department of Dermatology, Faculty of Medicine Siriraj Hospital, Mahidol University, Bangkok 10700, Thailand; 3 Division of Hematology, Department of Medicine, Faculty of Medicine Siriraj Hospital, Mahidol University, Bangkok 10700, Thailand

**Keywords:** extranodal natural killer/T-cell lymphoma, cutaneous lymphoma, interface dermatitis, granulomatous dermatitis, subcutaneous panniculitis

## Abstract

Background: Extranodal NK/T cell lymphoma, nasal type (ENK/T) with cutaneous involvement has various histopathological findings and diverse clinical manifestations. Methods: A retrospective study of cutaneous involvement of ENK/T lymphoma between 2006 and 2018 was conducted. Results: Twenty-two cases were eligible for this study. Twelve cases could be proven as secondary cutaneous involvement by ENK/T lymphoma, while the remaining could not be confirmed as primary cutaneous ENK/T lymphoma. The histopathological patterns included dermal and subcutaneous nodular infiltration pattern in 11/22 cases (50%), lobular panniculitis pattern in 6/22 cases (27.3%), interface dermatitis pattern in 4/22 cases (18.2%), and granulomatous dermatitis pattern in 1/22 case (4.5%). The median follow-up was 18.3 months. Overall, the one-year and five-year survival rates were 31.3% and 13.3%, respectively. Conclusions: A variety of histopathological patterns of cutaneous involvement by ENK/T lymphoma should be differentiated from other cutaneous lymphomas, dermatitis, and infection. When atypical medium or large-sized lymphoid cells are encountered within skin lesions, pathologists should realize these lesions can be ENK/T lymphoma, especially in cases with coexisting tumor necrosis or angioinvasion. A complete evaluation of the upper aerodigestive tract is mandatory to identify the occult primary site of ENK/T lymphoma before establishing primary cutaneous ENK/T lymphoma.

## 1. Introduction

Cutaneous involvement of extranodal NK/T-cell lymphoma, nasal type (ENK/T lymphoma) is a rare entity of cutaneous NK/T-cell lymphoma associated with Epstein–Barr virus (EBV) infection [1]. Clinical manifestations and histopathological characteristics are varied. The disease presents either primary or secondary cutaneous involvement, and solitary or multiple skin nodules can be found in the disease spectrum. Angiocentricity and angioinvasion with ischemic necrosis, as well as epidermotropism, are common histopathological findings [2]. Additionally, various microscopic patterns, including lobular panniculitis, granulomatous dermatitis, and interface dermatitis patterns have been reported in the cutaneous ENK/T lymphoma [3,4,5,6,7,8,9].

This study aimed to investigate the histopathological patterns of cutaneous ENK/T lymphoma, histopathological features, and clinical findings. In addition, clinical information, including head and neck examination, bone marrow involvement, and survival outcomes were investigated.

## 2. Materials and Methods

### 2.1. Case Materials and Histopathological Criteria

This study was approved by The Institutional Review Board, Faculty of Medicine Siriraj Hospital, Mahidol University (SIRB) (Si671/2561). A retrospective descriptive study of 30 patients diagnosed with cutaneous ENK/T lymphoma at the Department of Pathology, Faculty of Medicine Siriraj Hospital, from 2006 to 2018, was conducted. All sections were reviewed, and disease diagnosis was rendered if neoplastic cells were positive for EBV-encoded RNA in situ hybridization (EBER) and the presence of cytotoxic molecules, according to the WHO classification of skin tumors [1]. Regarding clinical manifestations, chronic EBV infection, mosquito bite reactions, and hydroa vacciniforme-like lymphoproliferative disorder were excluded from the study. Clinical history, laboratory investigations, treatments, and survival outcomes were recorded. Histopathological patterns and features were documented. These patterns included lobular panniculitis, interface dermatitis, granulomatous dermatitis, and dermal and subcutaneous nodular infiltration patterns. The lobular panniculitis pattern was defined as the infiltration of subcutaneous fat by atypical cells rimming around fat lobules. The interface dermatitis pattern required dermal infiltration by lymphoma cells and the overlying epidermis to show basal vacuolar changes with some necrotic keratinocytes. The granulomatous dermatitis pattern required the presence of granuloma. The dermal and subcutaneous nodular infiltration pattern required atypical lymphatic infiltration occupying more than a field of medium power magnification (×10 objective lens) or at least 2 mm diameter of atypical lymphocytic infiltration in the dermis or subcutaneous fat. Furthermore, histopathological features including angioinvasion, epidermotropism, and necrosis were listed. Bone marrow biopsies were reviewed and evaluated for bone marrow involvement in the disease, which is defined by a distinctive positive EBER among lymphoid cells or scattered medium- to large-sized cells with positive EBER. Findings in cases with scattered small lymphocytes with positive EBER were interpreted as minimal bone marrow involvement of the disease and no bone marrow involvement when there was no positive EBER.

### 2.2. Immunohistochemical Study and Epstein–Barr-Virus-Encoded Small RNA (EBER) In Situ Hybridization (ISH)

Immunohistochemistry data were re-evaluated by three pathologists (P.N., P.S., and S.S.). The panels of the immunohistochemical test included CD3 (LN10, 1:600, Novocastra, Leica Biosystems, Wetzlar, Germany), CD4 (SP35, ready to use, Ventana Medical Systems, Tucson, AZ, USA), CD5 (Cell Marque, 4C7, 1:100, Rocklin, CA, USA), CD8 (C8/144B, ready to use, Cell Marque, Rocklin, CA, USA), CD20 (L26, 1:2000, DAKO, Agilent, Santa Clara, CA, USA), CD30 (Ber-H2, 1:100, Cell Marque, Rocklin, CA, USA), CD56 (123C3.D5, 1:50, Cell Marque, Rocklin, CA, USA), βF1 (8A3, 1:20, Thermo Scientific, Waltham, MA, USA), γ-TCR (γ3.20, Thermo Scientific, Waltham, MA, USA), and TIA1 (TIA-1, 1:500, Biocare Medical, Pacheco, CA, USA). EBER ISH (Ventana Medical Systems, Tucson, AZ, USA) was also re-evaluated by the three pathologists.

### 2.3. Statistical Analysis

The continuous demographic data are presented as medians and ranges. The categorical demographic data are presented as percentages. The cumulative one-year and five-year survival rates were analyzed using a Kaplan–Meier analysis and are presented as Kaplan–Meier curves.

## 3. Results

### 3.1. Patient Characteristics

Out of a total of 30 patients with ENK/T lymphoma of the skin, 8 were excluded due to the lack of histopathological sections and paraffin blocks. Of the 22 recruited patients, 17 had complete clinical and histopathological data, and the remaining 5 had only histopathological sections and clinical history from pathology request forms. Of the 17 patients with complete data, 12 were classified with secondary cutaneous involvement by ENK/T lymphoma. Most of the primary sites of ENK/T lymphoma were the nasal cavity and/or nasopharynx. In three particular cases, each ENK/T lymphoma originated from the lacrimal gland (Case No. 5), uvula (Case No. 4), and spleen (Case No. 9). The remaining five patients were unclassified because they did not have enough information regarding the possible primary site of ENK/T lymphoma. We could not determine primary cutaneous ENK/T lymphoma in these five cases because of the lack of a tissue biopsy from the nasal cavity or nasopharynx to exclude occult lymphoma in these cases. Three of these five cases underwent nasal telescopy without a tissue biopsy. One case had a computerized tomography (CT) scan of the paranasal sinuses, but there was no tissue biopsy, and the other case did not undergo nasal telescopy or a CT scan. Demographic characteristics are demonstrated in Table 1.

**Figure 1:**
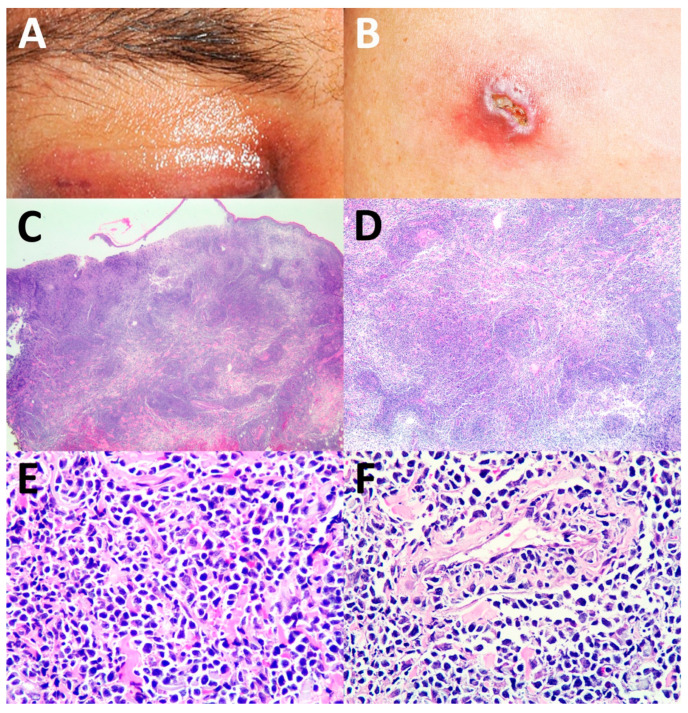
Dermal nodular infiltration pattern: (**A**) cellulitis-like lesion at the periorbital area; (**B**) abscess-like lesion of the skin; (**C**–**E**) dermal diffuse infiltration by atypical small to medium lymphoid cells (hematoxylin and eosin (H&E), original magnification ×40, ×100, ×400, respectively); (**F**) angioinvasion by atypical lymphoid cells (H&E, original magnification ×400).

**Figure 2:**
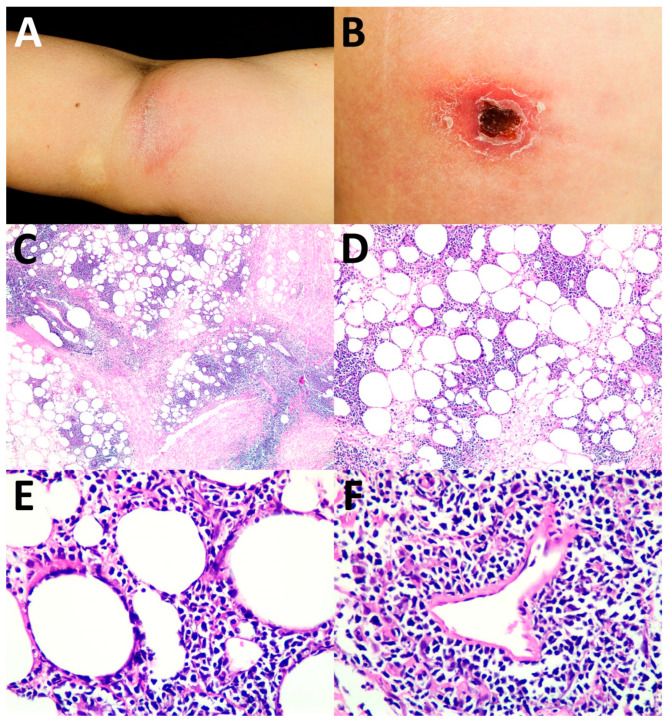
Lobular panniculitis pattern: (**A**) erythematous plaque and nodule without ulceration on right arm; (**B**) erythematous nodule with ulceration; (**C**) lobular infiltration by lymphoid cells (H&E, original magnification ×40); (**D**,**E**) periadipocytic rimming by atypical small-to-medium lymphoid cells (H&E, original magnification ×100 and ×400, respectively); (**F**) angioinvasion by atypical lymphoid cells (H&E, original magnification ×400).

**Figure 3:**
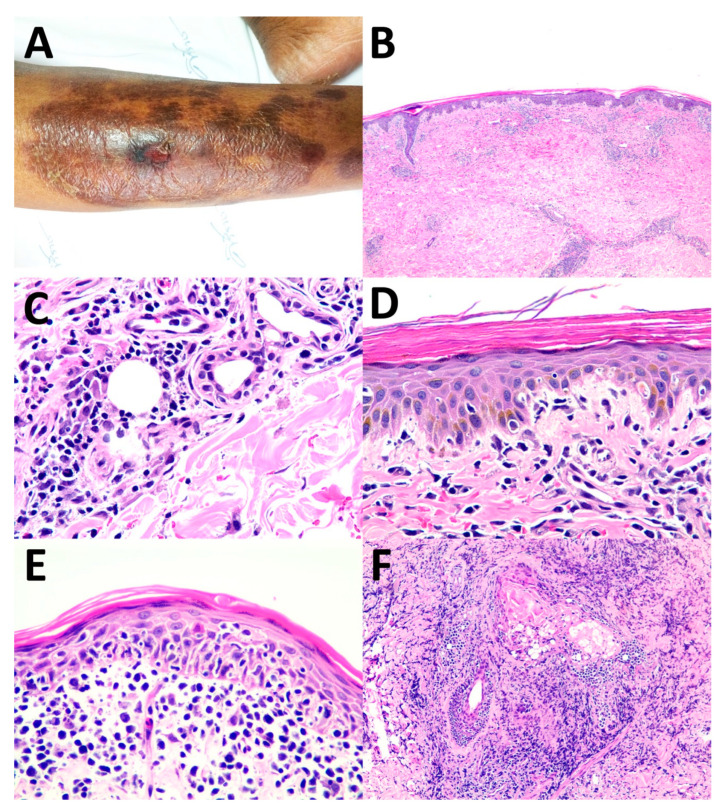
Interface dermatitis pattern: (**A**) multiple purplish plaques on right leg; (**B**) superficial and deep perivascular infiltration by small lymphocytes with overlying epidermal hyperkeratosis (H&E, original magnification ×100); (**C**) perieccrine infiltration by atypical small-to-medium lymphocytes (H&E, original magnification ×400); (**D**) basal vacuolar change and few necrotic keratinocytes (H&E, original magnification ×400); (**E**) presence of epidermotropism; (**F**) presence of folliculotropism (H&E, original magnification ×400).

**Figure 4:**
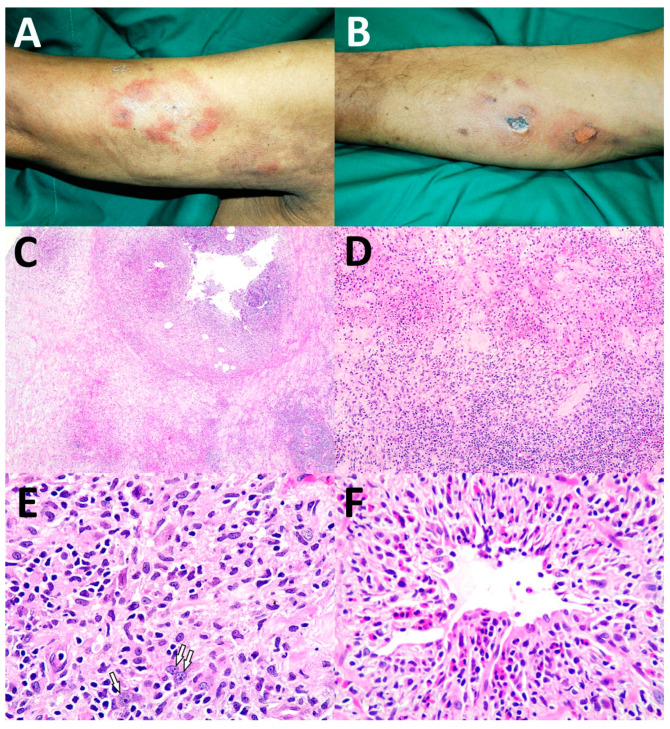
Granulomatous dermatitis pattern: (**A**) erythematous plaques on right arm; (**B**) nodules and plaques with ulcer on left leg; (**C**) lobular panniculitis with necrosis (H&E, original magnification ×100); (**D**) mixed infiltration of histiocytes, lymphocytes, and eosinophils (H&E, original magnification ×100); (**E**) granulomatous inflammation with few scattered atypical cells (white arrows) (H&E, original magnification ×400); (**F**) angioinvasion by atypical lymphoid cells and eosinophils (H&E, original magnification ×400).

Of the 17 patients who had complete data (Case No. 1–17), the cutaneous manifestations of ENK/T lymphoma varied (Table 2). Both solitary and multiple lesions could be found in secondary cutaneous involvement of ENK/T lymphoma. The locations of lesions included the head and neck region, trunk, abdomen, and extremities, with skin nodules being the most common. Interestingly, cellulitis-like (Figure 1A) and abscess-like lesions on the top of skin nodules (Figure 1B) were also found. Erythematous nodules with or without ulceration were present (Figure 2A,B). Papules and plaques could be seen as well (Figure 3A and Figure 4A,B).

### 3.2. Histopathology, Immunohistochemistry, and EBER ISH

The histopathological patterns are summarized in Table 3. The most common histological patterns were dermal and subcutaneous nodular infiltration (Figure 1C–E), which were found in 11 of 22 cases (50%). The other histopathological patterns included lobular panniculitis (Figure 2C–E) in six cases (27.3%), interface dermatitis (Figure 3B–D) in four cases (18.2%), and granulomatous dermatitis pattern (Figure 4C–E) in one case (4.5%). Angioinvasion (Figure 1F, Figure 2F, and Figure 4F) was found in 15 out of 22 cases (68.2%), tumor necrosis in 12 cases (54.5%), and epidermotropism and folliculotropism (Figure 3E,F) in 9 cases (40.9%). The tumor cell size in the majority of cases was medium-to-large atypical lymphoid cells; however, small tumor cell size was also noted.

In regard to the immunohistochemical study (Table 3), all the patients had positive EBER and cytoplasmic staining of CD3 without membranous staining. TIA-1 was positive in all the cases. Loss of CD56 reactivity was identified in one case. Few T-cell phenotypes (positive betaF1 and TCR-γ), aberrant expression of CD4 and CD8, and focal CD30 positivity were also present in lymphoma cells.

### 3.3. Bone Marrow Status

**Figure 5:**
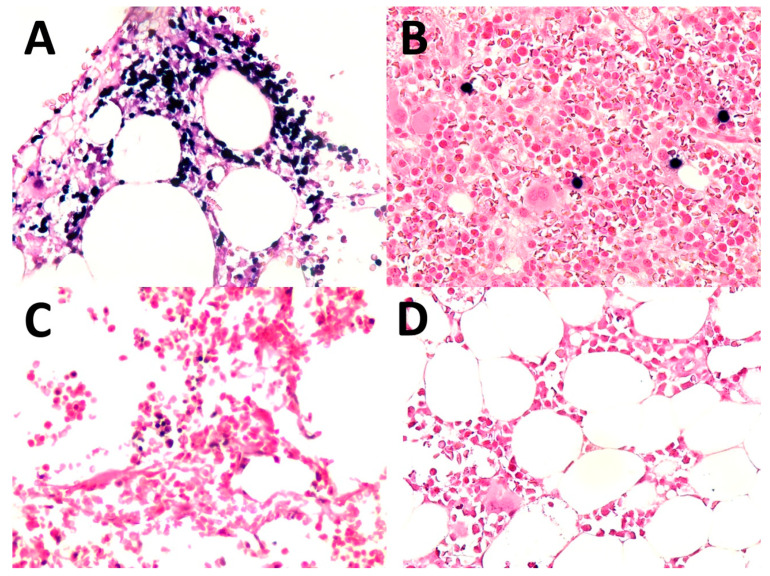
Bone marrow: (**A**) involved in ENK/T lymphoma, diffuse (EBV-encoded RNA in situ hybridization (EBER), original magnification ×400); (**B**) involved in ENK/T lymphoma, scattered cells (EBER, original magnification ×400); (**C**) scattered individual small lymphocytes with positive EBER (EBER, original magnification ×400); (**D**) negative for EBER+ cells (EBER, original magnification ×400).

Seventeen patients underwent bone marrow biopsies. Four of them (23.5%) had positive lymphoma cells in the bone marrow. Two out of the four had diffusely positive EBER (Figure 5), and the remaining two had positive EBER in scattered medium-to-large lymphoma cells (Figure 5B). In eight patients, there was the presence of scattered individual small lymphoid cells with positive EBER (Figure 5C) and seven of these eight patients had secondary cutaneous involvement by ENK/T lymphoma. A negative bone marrow biopsy was found in five patients (Figure 5D).

### 3.4. Treatment and Survival Outcome

**Figure 6:**
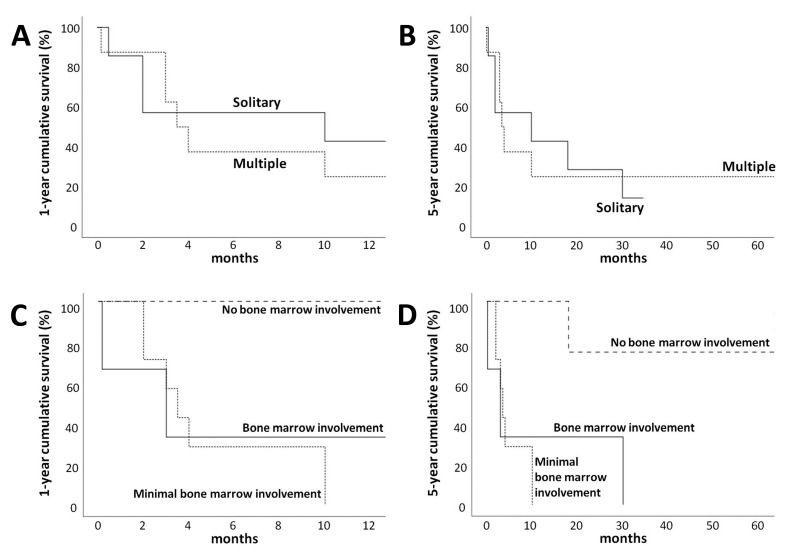
One-year and five-year cumulative survival rates: (**A**,**B**) comparison of cumulative survival between patients with solitary and multiple skin lesions; (**C**,**D**) comparison of cumulative survival between patients with bone marrow involvement, minimal bone marrow involvement, and no bone marrow involvement.

Of the 17 patients with complete clinical data, 14 underwent treatment for the disease and were in stage IV. The initial treatment in 14 patients consisted of combined chemotherapeutic agents. Four cases received concurrent radiotherapy. Complete response, partial response, and no response were observed in 57.1%, 28.6%, and 14.3% of the patients, respectively (Table 1). Six patients also experienced a relapse. Survival data were available for 16 patients. One case failed to follow up after six months of treatment. The median follow-up time was 18.3 months. Overall survival rates at 1 year and 5 years were 31.3% and 13.3%, respectively. The group of patients with solitary lesions tended to have a better one-year survival rate when compared with the group of patients with multiple lesions; however, the latter group had a better prognosis after 5 years (Figure 6A,B). The patient group who had the bone marrow involvement of ENK/T lymphoma and positive EBER in individual scattered small lymphoid cells showed poorer survival outcomes when compared with the group without bone marrow involvement (Figure 6C,D).

## 4. Discussion

Primary cutaneous ENK/T lymphoma is a rare type of lymphoma. Our study showed that most patients suffered from secondary involvement of ENK/T lymphoma primarily arising from the upper aerodigestive tract. We could not confirm primary cutaneous ENK/T lymphoma due to the lack of endonasal tissue biopsies in five patients who were labeled as unclassified, and in another five patients whose complete clinical profiles could not be retrieved. Random tissue biopsy at the nasal cavity and nasopharyngeal area has previously been recommended to determine occult lymphoma [10,11]. An imaging study of the nasal and nasopharyngeal regions using computerized tomography (CT) or positron emission tomography (PET) is used to investigate nasal and nasopharyngeal lymphoma in cases of extranasal ENK/T lymphoma; however, a major limitation is that an occult lymphoma can be missed [12]. A few studies have looked at the occult ENK/T lymphoma of the aerodigestive tract diagnosed after the initial presentation of ENK/T lymphoma in extranasal organs [13,14]. Thus, the presentation of cutaneous ENK/T lymphoma needs to exclude primary ENK/T lymphoma in the aerodigestive tract before establishing primary cutaneous ENK/T lymphoma.

Cutaneous manifestation is a common indicator of the extranasal involvement of ENK/T lymphoma that can be encountered either before or after the lymphoma of the aerodigestive system [14]. Skin lesions show various presentations and mimic infections such as cellulitis, abscess, and infective panniculitis, as shown in our study. Both single and multiple skin lesions are present in secondary involvement of ENK/T. In addition, various histopathological patterns of cutaneous involvement of ENK/T lymphoma mimic other variants of lymphoma, dermatitis, and the infectious process. Out of the total 22 patients in our study, the majority had dermal and subcutaneous nodular infiltration. Other patterns reported in patients were lobular panniculitis, interface dermatitis, and granulomatous dermatitis [3,5,7,9]. The dermal and subcutaneous nodular infiltration pattern can be seen in primary and secondary lymphoma and requires a complete clinical workup [15]. Tumor necrosis and vascular invasion were found in about half of all cases; however, these features can also be found in primary cutaneous γ/δ T-cell lymphoma or other lymphomas with aggressive behavior [16]. Although the clinical and histopathological findings of cutaneous ENK/T lymphoma vary, the detection of atypical lymphoid cells and angioinvasion along with immunohistochemistry and EBER ISH are practical for diagnosis.

The panel of immunohistochemistry is a helpful tool in the diagnosis of ENK/T lymphoma. Besides all the patients being EBER-positive, our study revealed that the majority of the cases were also positive for CD56, TIA-1, and cytoplasmic staining of CD3. These immunostainings are useful in the diagnosis of ENK/T lymphoma. The lobular panniculitis pattern of ENK/T lymphoma must be differentiated from subcutaneous panniculitis-like T-cell lymphoma (SPTCL) and gamma/delta T-cell lymphoma (GDTCL). A panel of immunohistochemistry and EBER ISH helps establish definite diagnoses. SPTCL typically shows positive results for CD8 and βF-1 positivity but negative results for CD56 and EBER. GDTCL commonly shows double-negative CD4 and CD8, positive CD56 and gamma TCR, and negative EBER. Positive CD56 and EBER can also be found in ENK/T lymphoma, with positive CD4 or CD8 in some cases. In any histologic section that has the periadipocytic rimming pattern with a doubtful diagnosis of lymphoma, EBER ISH should be included in addition to immunohistochemistry. In addition to immunohistochemistry, the cutaneous manifestations of SPTCL and GDTCL are not different from those of ENK/T lymphoma with a subcutaneous location. These lymphomas are presented with plaques and nodules. SPTCL mostly has an indolent clinical course, but the patients may develop the hemophagocytic syndrome, while GDTCL is clinically more aggressive, and it tends to have ulceration [17]. In our study, three out of the six cases of lobular panniculitis pattern (No. 7–9) were secondary ENK/T lymphoma. They clinically presented with solitary or multiple lesions with ulceration and necrosis in some lesions (Figure 2B), which may be indistinguishable from SPTCL and GDTCL except for the clinical evidence of primary ENK/T lymphoma, nasal type. Infectious conditions caused by mycobacterium or fungus should be initially excluded in any section showing a granulomatous reaction. This reaction has been found in various types of cutaneous lymphoma [18]. Some reports have demonstrated an association between granulomatous inflammation and cutaneous ENK/T lymphoma by mimicking infective panniculitis [3,5]. Atypical lymphoid cell infiltration in the background of reactive inflammatory cells (Figure 4E) and angioinvasion (Figure 4F) were also detected in our patient with the granulomatous dermatitis pattern of ENK/T lymphoma. 

Regarding the interface dermatitis pattern of ENK/T lymphoma found in our study, some previous studies have also shown interface alteration associated with cytotoxic T-cell lymphoma of the skin [19,20]. However, in the four cases with interface dermatitis patterns in our study, the lymphoma cells were negative for both CD4 and CD8. Basal vacuolar change accompanied by atypical lymphoid cell infiltration should raise concerns about cutaneous cytotoxic T-cell lymphoma or cutaneous involvement of ENK/T lymphoma. When a diagnosis of interface dermatitis is established, pathologists should look for atypia of lymphoid cells and other histopathological findings of the disease, particularly angioinvasion and epidermotropism.

Regarding the issue of epidermotropism, mycosis fungoides (MF) and primary cutaneous CD8+-aggressive epidermotropic cytotoxic T-cell lymphoma (CD8+ AECTL) were in the differential diagnosis when encountered with epidermotropism. MF is clinically slowly progressive from patches and plaques in sun-protected areas, which is different from ENK/T lymphoma [21]. Despite the lack of CD8+ lymphoma cells in the group with interface dermatitis patterns in this study, CD8+ AECTL should be in the differential diagnosis. Clinically, CD8+ AECTL presents with rapidly growing plaques and nodules with some ulcer and necrosis [17], quite similar to the clinical presentation of ENK/T lymphoma. Moreover, there were cases with CD8+ lymphoma cells in ENK/T lymphoma with T-cell origin [22]. Microscopic features of ENK/T lymphoma are diverse and the recognition of atypical lymphoid cells and angioinvasion, a broad panel of immunohistochemistry, especially TIA-1, CD56, the cytoplasmic stain of CD3, and EBER ISH, is important to establish a definite diagnosis of ENK/T lymphoma.

EBER ISH is a valuable tool in the evaluation of bone marrow involvement in ENK/T lymphoma [23]. A higher incidence of bone marrow involvement (23.5%) was found in our study, compared with 8–10% in another study [10]. The main reason for this difference was the high proportion of secondary cutaneous involvement, which indicated an advanced stage of the disease (stage IV) in our patients. The limitation of our study was the fact that EBER ISH was only performed in some cases without immunohistochemical staining. It is arguable whether cells in the bone marrow with a positive EBER stain are truly lymphoma cells or other reactive cells. Nevertheless, the authors evaluated the size of EBER-positive cells in the bone marrow and defined them as true medium-to-large lymphoma cells. Additionally, some individual scattered small lymphoid cells with positive EBER stains were also identified. The infiltration of these small cells in the bone marrow was designated as minimal bone marrow involvement, as proposed by a study of localized ENK/T lymphoma conducted by Lee et al. [23]. Their study and our study revealed that patients with minimal involvement of ENK/T lymphoma in the bone marrow had the same tendency of poorer overall survival outcomes as patients without bone marrow involvement (Figure 6C,D). These results suggested that detectable EBER-positive cells in the bone marrow may be correlated with poor survival outcomes.

In conclusion, various histopathological features and clinical presentations of cutaneous ENK/T lymphoma were demonstrated. ENK/T lymphoma should be differentiated from other types of lymphoma and dermatitis. EBER ISH is recommended in cases with a doubtful diagnosis of ENK/T lymphoma. Complete endonasal and nasopharyngeal examination, together with a blind biopsy of the regional tissue, should be performed in patients with cutaneous ENK/T lymphoma to exclude secondary cutaneous involvement of the disease. Regarding the clinical outcomes of the patients in this study, despite the small number of cases, the solitary or multiple lesions seemed to have no significant difference in survival rates, but the bone marrow involvement had some negative impact on survival.

## Figures and Tables

**Table 1:** Demographic data and clinical features.

Parameter		Analyzed Cases	
Age in years, median (range)		22	52 (25–87)
Sex, *n* (%)		22	
	Male		15 (68.2)
	Female		7 (31.8)
Presenting symptoms ^a^, *n* (%)		17	
	Fever		7 (41.2)
	Mass or nodule		4 (23.5)
	Nasal congestion		2 (11.8)
	Skin lesion		2 (11.8)
	Orbital cellulitis		2 (11.8)
	Others		4 (23.5)
B symptoms, *n* (%)		17	8 (47.1)
Cutaneous ulcer, *n* (%)		17	4 (23.5)
Visceral organ involvement ^b^, *n* (%)		17	6 (35.3)
Bone marrow involvement, *n* (%)		17	5 (29.4)
Treatment response, *n* (%)		14	
	Complete remission		8 (57.1)
	Partial remission		4 (28.6)
	No response		2 (14.3)
Relapse, *n* (%)		8	6 (75.0)
1-year survival, *n* (%)		16 ^c^	5 (31.3)
5-year survival, *n* (%)		15 ^d^	2 (13.3)

^a^ Some patients had more than one presenting symptom; ^b^ lung, liver, spleen, and brain; ^c^ one patient failed to follow up 6 months post-treatment; ^d^ one patient who survived was diagnosed with ENK/T lymphoma in less than 5 years.

**Table 2:** Clinical skin manifestations and histopathological patterns.

Case No.	Type	Distribution	Lesion	Location	Cutaneous Presentation	Histopathological Pattern
1	Secondary	Localized	Solitary	Hand, left	Ulcer	Dermal and subcutaneous nodular infiltration pattern
2	Secondary	Localized	Solitary	Upper eyelid	Cellulitis-like	Dermal and subcutaneous nodular infiltration pattern
3	Secondary	Generalized	Multiple	Extremities	Subcutaneous nodules	Dermal and subcutaneous nodular infiltration pattern
4	Secondary	Generalized	Multiple	Eyebrow, chin, and chest	Subcutaneous nodules	Dermal and subcutaneous nodular infiltration pattern
5	Secondary	Generalized	Multiple	Trunk and back	Plaques patches	Dermal nodular infiltration pattern
				Periorbital	Cellulitis-like	Dermal nodular infiltration pattern
6	Secondary	Generalized	Multiple	Arm, right	Dermal nodules	Dermal nodular infiltration pattern
7	Secondary	Localized	Solitary	Abdomen	Subcutaneous nodule	Lobular panniculitis pattern
8	Secondary	Localized	Solitary	Nasolabial fold	Skin nodule	Lobular panniculitis pattern
9	Secondary	Generalized	Multiple	Extremities	Plaques and subcutaneous nodules	Lobular panniculitis pattern
10	Secondary	Localized	Multiple	Leg, right	Papules and plaques	Interface dermatitis pattern
11	Secondary	Generalized	Multiple	Trunk and face	Papules	Interface dermatitis pattern
12	Secondary	Generalized	Multiple	Thighs and abdomen	Subcutaneous nodules	Granulomatous dermatitis pattern
13	Unclassified	Localized	Solitary	Shoulder, right	Skin nodule	Dermal and subcutaneous nodular infiltration pattern
14	Unclassified	Generalized	Multiple	Trunk, upper and lower extremities	Skin nodules	Dermal and subcutaneous nodular infiltration pattern
15	Unclassified	Generalized	Multiple	Upper and lower extremities	Nodules	Dermal and subcutaneous nodular infiltration pattern
16	Unclassified	Localized	Solitary	Arm, right	Cellulitis-like	Lobular panniculitis pattern
17	Unclassified	Localized	Solitary	Abdomen	Subcutaneous nodule	Lobular panniculitis pattern

**Table 3:** Histopathological patterns and immunohistochemistry.

Case No.	Tumor Necrosis	Vascular Invasion	Epidermotropism	Tumor Cell Size	CD3	CD4	CD5	CD8	CD20	CD30	CD56	TIA-1	BetaF1	TCR-γ	EBER
Dermal/Subcutaneous nodular and diffuse infiltration pattern															
1	+	+	+	M to L	+	-	NA	-	-	NA	-	+	-	-	+
2	-	-	-	M to L	+	NA	NA	NA	NA	NA	-	+	NA	NA	+
3	+	+	-	Mto L	+ ^#^	NA	-	-	NA	+ *	+	+	NA	NA	+
4	-	+	-	M to L	+	-	+ *	-	-	+	+	+	-	NA	+
5	-	-	-	M to L	+	-	-	-	-	+ *	+ ^#^	+	NA	NA	+
6	-	+	+	L	+	-	-	-	-	+ *	+	+	-	-	+
13	-	-	-	L	+	-	-	-	-	NA	+ ^#^	+	-	-	+
14	-	-	+	S to M	+	-	-	-	-	+ *	+	+	-	NA	+
15	+	+	-	L	+	-	-	-	-	+ *	+	+	-	NA	+
X1	+	+	-	L	+	NA	-	NA	-	+	+	+	-	NA	+
X2	+	-	-	L	+	-	-	-	-	NA	+	+	-	NA	+
Lobular panniculitis pattern															
7	+	+	-	M to L	+	NA	-	NA	-	NA	+	+	NA	NA	+
8	+	-	+	M with L	+	+	+	+	-	NA	+ *	+	+ *	+	+
9	+	+	-	L	+	-	NA	-	-	+	+	+	NA	-	+
16	-	+	+	S, M, L	+	-	NA	+	-	-	+	+	+	-	+
17	+	+	-	M to L	+	NA	NA	NA	-	NA	+	+	+	NA	+
X3	+	+	-	M to L	+	-	NA	NA	-	+ *	+	+	NA	NA	+
Interface dermatitis pattern															
10	-	+	+	M to L	+	-	+	-	NA	+ *	+	+	NA	NA	+
11	-	+	+	L	+	-	-	-	-	+	+ ^#^	+	NA	NA	+
X4	-	-	+	S to M	+	-	NA	-	-	NA	+	+	NA	-	+
X5	+	+	+	M to L	+	-	-	-	-	+	+	+	-	NA	+
Granulomatous dermatitis pattern															
12	+	+	-	L	+	-	-	+	-	+	+	+	NA	NA	+

^#^, Faintly positive; *, focal positive; -, negative; +, positive; L, large; M with L, medium with occasional large; M, medium; NA, not available; S, small; Patient No. X1-X5 had unavailable clinical data.
